# Assessing current and future available resources to supply urban water demands using a high-resolution SWAT model coupled with recurrent neural networks and validated through the SIMPA model in karstic Mediterranean environments

**DOI:** 10.1007/s11356-024-34404-5

**Published:** 2024-07-24

**Authors:** Antonio Jodar-Abellan, Miguel Ángel Pardo, Seyed Babak Haji Seyed Asadollah, Ryan T. Bailey

**Affiliations:** 1 Soil and Water Conservation Research Group, Centre for Applied Soil Science and Biology of the Segura, Spanish National Research Council (CEBAS-CSIC), Campus de Espinardo 30100, P.O. Box 164, Murcia, Spain; 2 Department of Civil Engineering, University of Alicante, Alicante, Spain; 3 Department of Environmental Resources Engineering, State University of New York College of Environmental Science and Forestry (SUNY ESF), 1 Forestry Dr, Syracuse, NY 13210 USA; 4 Department of Civil and Environmental Engineering, Colorado State University, Fort Collins, USA

**Keywords:** Hydrological modelling, Wavelet analysis, Groundwater exploitation, Deep learning algorithms, Semiarid karstic watersheds

## Abstract

Hydrological simulation in karstic areas is a hard task due to the intrinsic intricacy of these environments and the common lack of data related to their geometry. Hydrological dynamics of karstic sites in Mediterranean semiarid regions are difficult to be modelled mathematically owing to the existence of short wet episodes and long dry periods. In this paper, the suitability of an open-source SWAT method was checked to estimate the comportment of a karstic catchment in a Mediterranean semiarid domain (southeast of Spain), which wet and dry periods were evaluated using box-whisker plots and self-developed wavelet test. A novel expression of the Nash–Sutcliffe index for arid areas (ANSE) was considered through the calibration and validation of SWAT. Both steps were completed with 20- and 10-year discharge records of stream (1996–2015 to calibrate the model as this period depicts minimum gaps and 1985–1995 to validate it). Further, SWAT assessments were made with records of groundwater discharge and relating SWAT outputs with the SIMPA method, the Spain’s national hydrological tool. These methods, along with recurrent neural network algorithms, were utilised to examine current and predicted water resources available to supply urban demands considering also groundwater abstractions from aquifers and the related exploitation index. According to the results, SWAT achieved a “very good” statistical performance (with ANSE of 0.96 and 0.78 in calibration and validation). Spatial distributions of the main hydrological processes, as surface runoff, evapotranspiration and aquifer recharge, were studied with SWAT and SIMPA obtaining similar results over the period with registers (1980–2016). During this period, the decreasing trend of rainfalls, characterised by short wet periods and long dry periods, has generated a progressive reduction of groundwater recharge. According to algorithms prediction (until 2050), this declining trend will continue reducing groundwater available to meet urban demands and increasing the exploitation index of aquifers. These results offer valuable information to authorities for assessing water accessibility and to provide water demands in karstic areas.

**Supplementary Information:**

The online version contains supplementary material available at 10.1007/s11356-024-34404-5.

## Introduction

Water resources from karst aquifers make up a key part of the population’s drinking water supply, both in a global and in a European context (Hartmann et al. 2013; Derdour et al. 2023). In line with Kalhor et al. (2019) and Ntona et al. (2023), around 20–25% of world’s inhabitants either live on or achieve their water supply from karst environments. This ratio becomes greater in certain regions as Europe where it is close to 35% (Kalhor et al. 2019) because of soluble carbonate rocks covering an important area of Europe, principally in the southern, eastern and western part of this continent (Malagò et al. 2016; Tohuami et al. 2013). Some limestone geological units spread large territories in the south of France, the Balkan region, Italy, Turkey, numerous islands of the Mediterranean (e.g. Majorca, Crete, Sicily) and Spain, where karst groundwater extends over a surface of 54,628 km^2^ (11% of the whole country). Thus, the physical geography and geology of Mediterranean watersheds are influenced by karst processes whose aquifers and springs provide groundwater resources to urban, agricultural or industrial demands in their vicinity (Malagò et al. 2016; Valdes-Abellan et al. 2020).

Karstic environments provide a singular geological morphology and landscape made by the melting activity of rainwater on soluble carbonate materials as limestone mainly but besides dolomite, marble and/or magnesite (Tohuami et al. 2013; Al Khoury et al. 2023). Dissolution processes (“karstification”) lead to the creation of springs, sinkholes, sinking streams and caves, which make up the representative features of a karst structure. As a result, these systems depict various types of porosity with a completely hydraulic behaviour (Kalhor et al. 2019; Nerantzaki et al. 2020). As karstification in a region advances, groundwater current into the karst aquifer progresses from low flow in a connected fractured system to high flow more concentrated within the greatest conduits such as numerous great pipes, interconnected cavities and caves (Malagò et al. 2016). The downstream culmination of a karst structure frequently consists of a spring by which the underground channels spread the surface of the terrain as a located discharge from a long system of groundwater channels (Nguyen et al. 2020; Vallejos et al. 2015).

In this context, large/regional scale hydrologic and hydrochemical models, together with models built to be used at the aquifer scale, are required to manage present and forthcoming water reservoirs (Hartmann et al. 2013; Alcalá et al. 2018). Regional-scale models with a suitable discretisation of the basin and subbasins have been shown to properly account for the spatial heterogeneity and provide appropriate water-yield predictions in karstic regions (Wang and Brubaker 2014; Mo et al. 2023). Among basin-scale hydrological tools, lumped and distributed models must be differentiated. Distributed models divide each study domain into a grid with two or three dimensions being water flow solved with physical equations in each grid cell (Hunink et al. 2017; Zhu et al. 2023). In these cases, a parameterisation of hydraulic conductivities, geometry/site of channels, porosities and canal roughness, among others are necessary with great spatial resolution accuracy. However, this information is often scarce in karst areas (Nguyen et al. 2020). This difficulty to get the complete characterisation for the whole karstic domain leads to applying distributed models to reproduce the hydrological behaviour of these sites using estimated values to represent physically based parameters (Mostofa-Amin et al. 2017). In contrast, lumped models estimate physic processes by means of equation clusters which transport water, among other variables, from the input to the output of the karst area. In regional models, the scale of the karst used to vary from the entire watershed to subbasins and/or subpolygons (which depict subbasins disaggregated into many small units). Lumped methods allow identifying recharge volume and conduits storage into the soil profile and, in the epikarst, water flow via discharge or sinking streams through several springs (Nikolaidis et al. 2013; Kalhor et al. 2019). Usually, lumped models consider more processes than distributed models and permit running over longer periods of time, as well as during high-flow and low-flow periods (Wei et al. 2019; Jin et al. 2023). The ease of executing lumped tools and their moderately minor computational time are important advantages, jointly with automatic calibration of sensitive parameters from the model through inverse modelling (Abbaspour et al. 2015; Palacios-Cabrera et al. 2022). These features are important in areas with scarce parameter measures (Al Khoury et al. 2023). Because of the above conditions, currently lumped models are often preferred to be used in areas with singular features as karstic sites (Hartmann et al. 2013; Kumar et al. 2023).

As stated in Mostofa-Amin et al. (2017) and Nikolaidis et al. (2013), the most relevant scientific studies accomplished in karstic basins can be grouped in (i) transport flow by Manning’s expression, (ii) groundwater models with distributed parameters such as MODFLOW (Harbaugh 2005; Mcdonald and Harbaugh 1984) that use effective hydraulic conductivity to represent conduits and other karstic features, (iii) groundwater reservoir depicted as a storage, (iv) distributed hydrological tools which depict channel routing and (v) semidistributed hydrological models whose units (subbasins) interact with independency. In this sense, it should be noted the semi-distributed (lumped) Soil and Water Assessment Tool (SWAT) (Arnold et al. 1998), which includes most of the mentioned modelling approaches and often is used for studies that assess climate and land use change (Samavati et al. 2023; Martínez et al. 2021; Aibaidula et al. 2023; Kumar et al. 2023), land-administration practices on water supply (Mostofa-Amin et al. 2018; Pandi et al. 2022; Jin et al. 2023), flash flood approaches (Jeong et al., 2010; Jodar-Abellan et al. 2019b; Tefera and Ray 2023), agricultural chemical yields and sediment transport of ungauged and gauged catchments (Rouholahnejad et al. 2014; Jimeno et al. 2022), groundwater assessment when linked with MODFLOW (Sisay et al. 2023; Bailey et al. 2016; Wei et al. 2019; Molina et al. 2019), etc. SWAT is the most often-utilised semi-distributed hydrologic tool (Aloui et al. 2023; Gassman et al. 2014; Tan et al. 2020) and has been considered to simulate hydrological dynamics in karstic sites (Malagò et al. 2016; Nerantzaki et al. 2020), estimating, for instance, pollutant concentrations and daily loads (Baffaut & Benson 2009; Mostofa-Amin et al. 2017) or the variability of both surface runoff and karst flow (Nikolaidis et al. 2013). SWAT can work coupled with others methods such as the chloride mass balance (CMB) to enhance the streamflow modelling (Senent et al. 2020). Some researchers have improved the model in karstic regions by introducing new algorithms (Wang and Brubaker 2014; Nguyen et al. 2020) to get data of percolation (Sakaguchi et al. 2014) or to characterise land cover conditions (Wang et al. 2018). However, around the world, the number of scientific studies applying SWAT in karstic environments is still limited regarding other areas such as detritic watersheds (Ollivier et al. 2020; Al Khoury et al. 2023), being even more relevant study the suitability of the SWAT model in karstic sites with scarce water resources.

Principal goals of the present work were as follows: (1) Implement and validate the SWAT code in a Mediterranean karstic basin (SE Spain) characterised by semiarid climatic conditions and by several springs that both release groundwater in specific locations and provide water resources to be infiltrated in others. Thus, daily precipitation for 1980–2016 was analysed to find the length and distribution of dry and wet periods, together with their frequencies (rainfall cycles), with box-whisker plots and with an own developed wavelet analysis. SWAT results were tested with the following: (i) monthly flow records through sensitive and uncertainly analysis (calibration and validation) to the period 1985–2015, (ii) daily natural groundwater sources (validation) over 7 years (2007–2014) and (iii) the Spanish national hydrologic modelling performed in this country within the distributed SIMPA model (MIMAM 2000), validation process, at a yearly scale to 36 years (1980–2016). In particular, the regional scale SWAT model was chosen due to the following: (i) the limited groundwater information in the karst study area; (ii) the possibility of restrict parameters uncertainty, especially noted at groundwater scale, within calibration and validation because of numerous flow records (inverse modelling); and (iii) the suitability of SWAT to assess key issues identified at the study basin over the long period, related with water resources available to supply close populations (urban demands), with water quality problems and land use changes. (2) Since urban water demands involved in the watershed are supplied mainly with groundwater (Valdes-Abellan et al. 2020), aquifers recharge from SWAT and SIMPA, at yearly scale, were considered to assess their availability to supply these populations during the period with registers (1980–2016). Next, groundwater recharge was estimated, until 2050, within recurrent neural network algorithms (Asadollah et al. 2021; Li et al. 2023) considering recharge series of SWAT, in each hydrologic response unit of the basin, as inputs to the algorithms. As well, this current and predicted offer of groundwater was related to urban water demands (annual abstractions from aquifers) within the exploitation index (Jodar-Abellan et al. 2019a).

Based on the above-mentioned objectives, we pretend to address the great number of scientific unanswered questions in this matter: Could the open SWAT code reach satisfactory results in karstic semiarid environments during calibration and validation routines? How will this model work in hydrological environments with long dry periods and short heavy precipitation events? Can SWAT reproduce adequately hydrological dynamics of the catchment system with the presence of several springs spread along the basin? Can recurrent neural networks be successfully coupled with SWAT outputs?

## Studied watershed

**Fig. 1:**
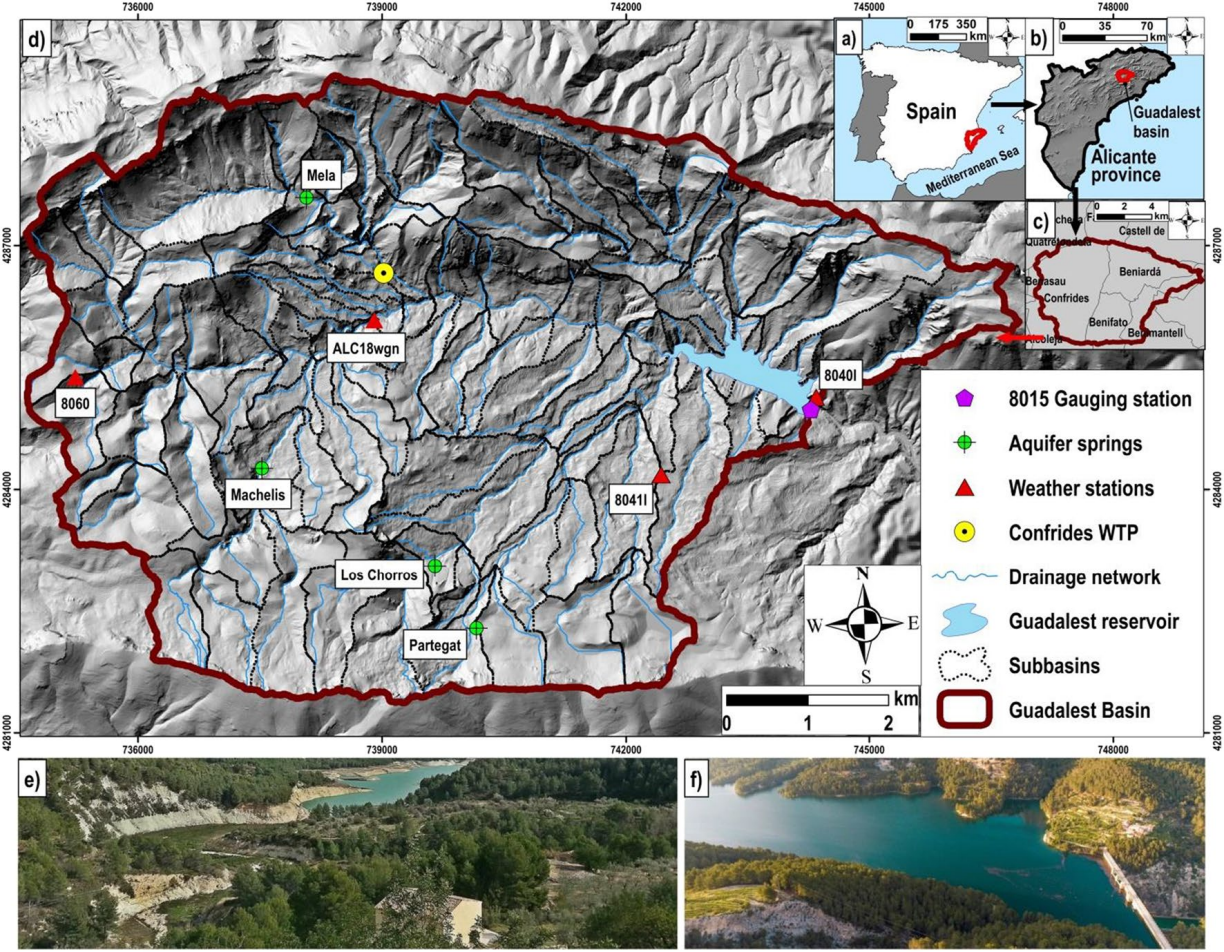
Study area: the *Guadalest* watershed in the context of **a** Spain, **b** *Alicante* province, **c** main municipalities, **d** regional framework, pictures of **e** the basin, and **f** the *Guadalest* reservoir during the fieldwork

This work was performed in the *Guadalest* catchment, a Mediterranean karst watershed of 62 km^2^ placed in the semiarid part of the Alicante region (southeast of Spain, Fig. 1). This area belongs to the eastern portion of the Baetic mounts, Prebaetic mountain chain (DPA-IGME 2015). The watershed is located 13.7 km from the coastline (Fig. 1) and shows a mean altitude of 864 m above sea level (m.a.s.l), having the maximum altitude 1556 m.a.s.l and the minimum 363 m.a.s.l. (DGTP 2009). As stated in the digital elevation model (DEM) from CNIG (2023), steep slopes (around 30–40%), ravines and ephemeral streams are identified in most parts of the basin (Fig. 1).

Weather stations with daily series provided by the Meteorology National Administration of Spain (AEMET) show, in the basin (Fig. 1), annual average temperatures of 14.2 °C and rainfall of 568 mm/year during the period 1980–2016. Watershed climate is classified as Mediterranean, warm and dry summer along with moderate winter (Valdes-Abellan et al. 2017; AEMET 2023).

**Fig. 2:**
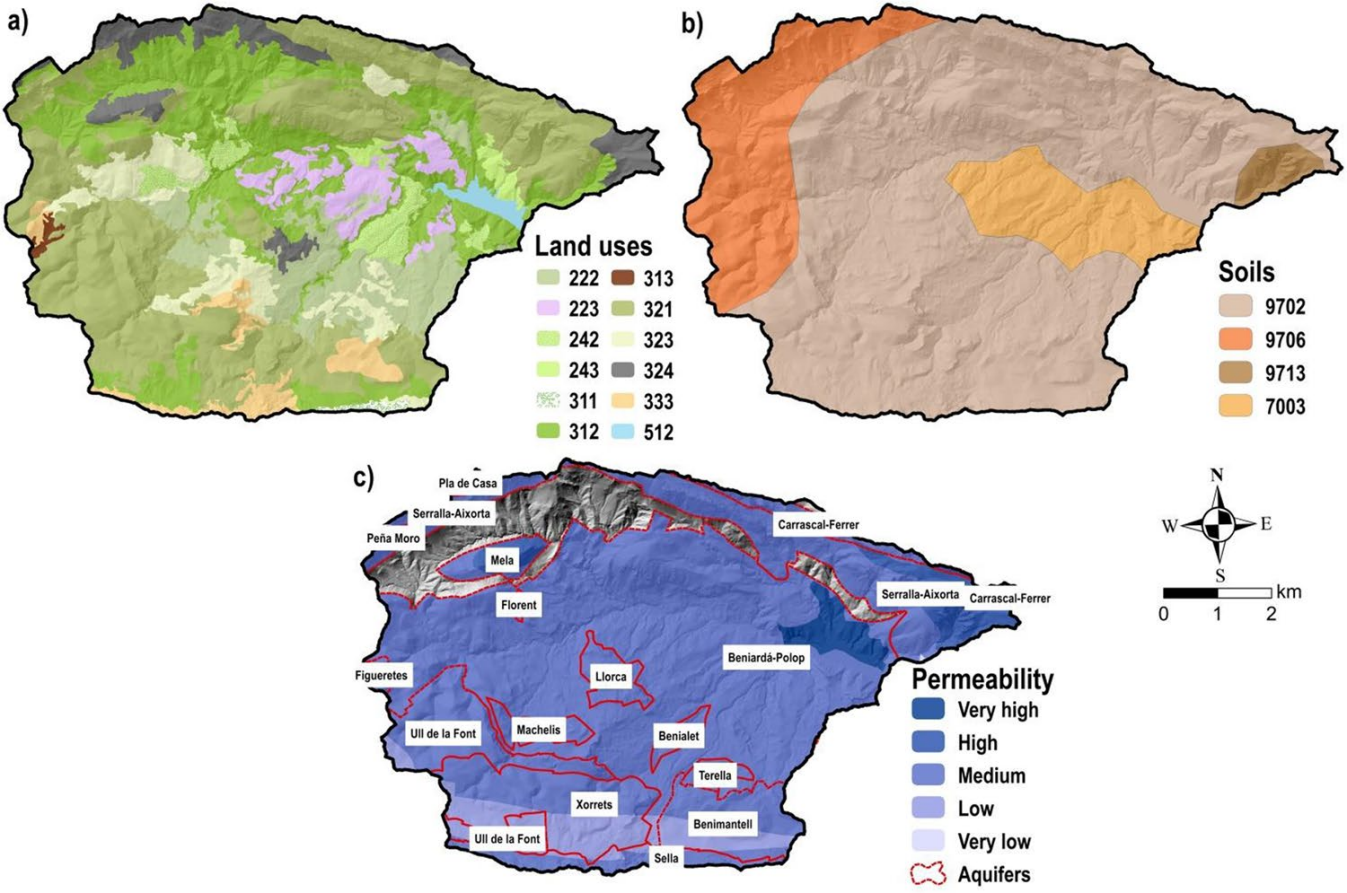
The *Guadalest* basin showing cartography relative to **a** land uses, **b** soils (pedology), and **c** aquifers along with its permeability classes

According to land use cartography from the CORINE Land Cover (CLC) database (CNIG 2023), no urban uses are recognised (Fig. 2a) being agricultural and forest the dominant land uses, with 18.6% and 80.6%, respectively, of the catchment area. Subcategories of these dominant land uses are shown in Table 1 and their spatial distribution in Fig. 2a. Likewise, surface of the *Guadalest* reservoir is classified as water body (Table 1 and Fig. 2a). Harmonized World Soil Database register’s (HWSD 2023) show that Lithic Leptosols with inclusions of Calcaric Cambisols is the main soil in the basin, followed by Calcaric Cambisols with inclusions of Calcaric Regosols, water bodies and Calcaric Cambisols alongside insertions of Chromic Luvisols. These soil classes receive, respectively, the codes: 9702, 9706, 7003 and 9713 (Fig. 2b) following the nomenclature of HWSD (2023) and Nachtergaele et al. (2012).

**Table 1:** Land cover categories from CORINE Land Cover (2018 period) converted into the equivalent categories of SWAT and Manning roughness factors, at the overland scale, in line with the Engman (1986) classes

CLC code	Land use class	SWAT code	Land use class	Overland Manning’s roughness (n_ov_)	
				Value	Category
222	Fruit trees and berry plantations	ORCD	Orchard	0.60	Rangeland
223	Olive groves	OLIV	Olives	0.22	Conventional tillage
242	Complex cultivation patterns	AGRR	Agricultural land-row crops	0.22	Conventional tillage
243	Land principally occupied by agriculture, with significant areas of natural vegetation	AGRL	Agricultural land-generic	0.16	Shortgrass prairie
311	Broad-leaved forest	FRSD	Forest-deciduous	0.50	Rangeland
312	Coniferous forest	FRSE	Forest-evergreen	0.60	Rangeland
313	Mixed forest	FRST	Forest-mixed	0.60	Rangeland
321	Natural grassland	RNGE	Range-grasses	0.24	Dense grass
323	Sclerophyllous vegetation	SWRN	Southwestern arid range	0.11	Chisel plow
324	Transitional woodland-shrub	RNGB	Range-brush	0.60	Rangeland
333	Sparsely vegetated areas	BARR	Barren	0.12	Chisel plow
512	Inland waters/water bodies	WATR	Water	0.01	-

Lithology of the watershed comprises materials from the Mesozoic (Triassic and Cretaceous periods) and the Cenozoic, mainly from the Quaternary and Tertiary ages (DPA-IGME 2009). Concerning the geomorphology, landforms were formed by limestone, dolomite, gypsum, conglomerate and sandstone which compound the karst environment in the basin and present a permeability varying from medium to high and very high (Fig. 2c). According to IGME (2021), certain areas, such as the south of the basin, depict deposits of marls and clays with low and very low permeability. These materials constitute 16 aquifers in the catchment (Fig. 2c), which, due to its karstic nature, shows a rapid flow response to rainfalls collected in the basin through its natural springs (Tohuami et al. 2013). Figure 1 illustrates the main aquifers springs, according to the registered data period, identified in the watershed.

The *Guadalest* watershed is close to the natural hydrologic regime, mainly, since population is scarce and disperse (DPA-IGME 2015). Around 750–770 inhabitants are registered and distributed in 6 small localities (INE 2023), named as their municipalities: *Castell de Guadalest*, *Benifato*, *Benimantell*, *Beniardá*, *Abdet* and *Confrides* (Fig. 1). These inhabitants are supplied principally by groundwater and scarcely by surface resources collected in the *Guadalest* reservoir. This reservoir, placed at the basin outlet (Fig. 1), can store a maximum volume of 13 hm^3^, although an average reserve of 7.8 hm^3^ has been identified during the last decade (CHJ 2023). As described by CEDEX (2023), the *Guadalest* reservoir receives water from the *Confrides* wastewater treatment plant (WTP), which pours its treated sewage (108.3 m^3^/day) to the drainage network (Fig. 1).

## Methodology

### The SWAT method

Hydrological predictions were achieved through SWAT (Arnold et al. 2012), a semi-distributed, time-continuous hydrological code that works at a catchment scale (Aloui et al. 2023; Winchell et al. 2013). This study was done with SWAT version 2012.10.4.21, as a plug-in of ArcGIS (ArcSWAT). The following hydrological input data and operations were used to build this physically based model.

#### Spatial information

The *Guadalest* watershed and its subbasins were defined using a DEM with 5 m × 5 m of spatial resolution (CNIG 2023). A pre-filter of the DEM was done, with GIS tools, in order to eliminate peaks and sinks (Tarboton et al. 1991; Leong-Tan et al. 2023). Hence, a correct definition of streams, basin and subbasins were ensured avoiding also the creation of a discontinuous drainage system. To enhance the mentioned definition, the layer of drainage channels (scale 1:25,000), which modifies Pfafstetter river categories (MAPAMA 2023), was coupled in SWAT. Thus, suitable flow connexions among subbasins were reached. The basin output was fixed at the *Guadalest* reservoir gauge station, and 95 subbasins were achieved (Fig. 1). This large number in a basin of 62 km^2^ is a consequence of the reduced threshold fixed in SWAT within the flow accumulation and direction process from the DEM (de Brouwer et al. 2017; Eekhout et al. 2024). According to our aims, it was essential to get a high spatial resolution in the basin.

Subbasins were divided in hydrologic response units (HRUs), the maximum spatial accuracy of SWAT (Abbaspour et al. 2015). In each subbasin, HRUs were reached by intersecting the slope, land cover and soil (pedology) data (Leong-Tan et al. 2023). The CLC database was considered to acquire land coverage, in this case to the most current scenario (2018) and with 1:100,000 as scale (CNIG 2023), being its land cover categories reclassified into land use types of SWAT (Table 1). Slope cartography, created from the previous DEM, was separated into two categories (0–3% and > 3%) as stated in the 5.2-IC Spanish regulation (BOE 2016). Regarding soil features, SWAT depicts soil classification according to the soil taxonomy from the US Agriculture Department (Neitsch et al. 2011; Arnold et al. 2012), whereas most of Spanish soil cartography were made following the FAO90/UNESCO soil taxonomy (Gomariz and Alonso 2018). Therefore, it is not possible to perform a direct soil reclassification as was done in the land use coverage. Thus, a novel database of soils was generated in this work using the raster soil layer of HWSD (2023), which has been implemented in Spain with trustworthy results (Malagò et al. 2015). HWSD (2023) shows dominant and soil inclusions divided in two layers: a topsoil (the first 30 cm of the terrain) and a bottom soil layer (among 30 and 100 cm). From the HWSD (2023) soil raster and within the Soil–Water Features software (SPAW 2023; Saxton and Rawls 2006), several soil parameters required by the SWAT soil database were calculated for the *Guadalest* basin following the work routing of Gomariz and Alonso (2018). Because of the accomplished land use, slope and soil intersections, a total of 732 HRUs were obtained in the basin.

#### Meteorological data

Observed meteorological data were gathered at a daily scale from the 8060, ALC18wgn, 8040I and 8041I weather stations (Fig. 1), supported by AEMET (2023). These stations span the length of the watershed and contain at least 30 years of daily rainfall, maximum and minimum temperature registers (*Tmax* and *Tmin*) and minor gaps, permitting to obtain the principal climatic characteristics in this area. Along with rainfall, *Tmax* and *Tmin* data, SWAT requires daily meteorological series of wind speed, solar radiation and relative humidity (Winchell et al. 2013). However, since our basin does not show daily registers of these variables (AEMET 2023), the SWAT weather generator was used to build their daily series (Sharpley and Williams 1990; Neitsch et al. 2011). Registers of the ALC18wgn station (Fig. 1) were considered to calculate meteorological parameters, with monthly time step, required to run this generator. In particular, the other meteorological stations do not possess initial information to calculate these parameters. Daily potential evapotranspiration was modelled in SWAT, within the Hargreaves equation, by using the described weather data (Hargreaves et al. 1985; Samani 2000).

#### Precipitation patterns

Daily precipitation series from 1980 to 2016 were assessed to determine the duration of dry and wet periods, together with their frequencies (rainfall cycles). The length and distribution of both periods were obtained through box-whisker plots, which divide the observed values within the median quartiles (25th and 75th percentile) and the outliers (Krzywinski and Altman 2014). According to studies developed in semiarid and arid regions as Valdes-Abellan et al. (2017), Santos et al. (2017) and Sillero et al. (2021), days were classified as dry or wet if daily precipitation exceeds or does not exceed 1 mm.

A wavelet test (Grinsted et al. 2004; Jevrejeva et al. 2003) was implemented in order to identify the magnitude and presence of rainfall cycles. In particular, continuous wavelet transform (CWT), considering Morlet as the main wavelet (Pla et al. 2020), was used to recognise signal frequencies of rainfall data. This analysis was accomplished with the environmental wavelet code proposed by Pla et al. (2020), which was based in the MATLAB toolbox of Grinsted et al. (2004).

#### Propagation, flow velocity and surface runoff

Rainfall-runoff conversion was performed within the curve number procedure of the Soil Conservation Service (SCS 1986; Neitsch et al. 2011) for the moisture condition II (CNII). Main data required by this method (land cover, soil permeability, antecedent soil water features, etc.), together with slope adjustments (Williams 1995), belong to the previous cartography coupled in SWAT. Therefore, it is considered as an appropriate method to achieve main purposes of this study. Within the CNII method, calculating the retention variable (*S*) for a day (mm), a 0.99 value was fixed as the weighting factor (*cncoef*) to estimate the retaining coefficient to daily curve numbers based on plant evapotranspiration (Arnold et al. 2012). This value was established in view of certain experiences performed in the southern of Spain (Gomariz et al. 2018; Palacios-Cabrera et al. 2022).

Parameters for routing water through channels and overland flow were obtained using Muskingum and roughness Manning methods. Both obtained suitable results in SWAT studies achieved in semiarid and arid Mediterranean regions (Hartmann et al. 2013; Kehew et al. 2010; Martínez and Conesa 2020). Flow propagation was addressed across the canals net by the Muskingum River routing model (Overton 1966; Cunge 1969) which principal formulation is presented in Eq. (1):

1 $${V}_{stored}=K\bullet (X\bullet {q}_{in}+(1-X)\bullet {q}_{out})$$

where *V*_*stored*_ is the reservoir volume (m^3^ H_2_O), *K* is the reservoir time constant for reaches (s), *q*_*in*_ is the inflow (m^3^/s), *q*_*out*_ is the outflow (m^3^/s) and *X* is the weighting coefficient that adjusts the influence of *q*_*in*_ and *q*_*out*_ in each reach. As stated by Ntona et al. (2023) and Neitsch et al. (2011), *X* received a value of 0.2 in the *Guadalest* channels. *K*, calculated according to Muskingum equation, is affected by two calibration coefficients which regulate the effect of peak flow (high runoff rates) and low flow (*K*_*co1*_ and *K*_*co2*_). Since channel features of this watershed, *K*_*co1*_ and *K*_*co2*_ received values of 0.25 and 0.75. As well, the velocity and flow rate in the chief and tributary channels and at the overland scale, for a given time step (Eq. 2 and Eq. 3), were accomplished with the Manning’s method for constant flow (Eekhout et al. 2024; Manning et al. 1977):

2 $${v}_{ov}=\frac{0.005\bullet {L}_{slp}^{ 0.4}\bullet {slp}^{0.3}}{{n}_{ov}^{ 0.6}}$$

3 $${v}_{c}=\frac{0.317\bullet {area}^{0.125}\bullet {slp}_{ch}^{ 0.375}}{{n}_{c}^{ 0.75}}$$

where *v*_*ov*_ is the flow speed (m/s) in the overland, *L*_*slp*_ is the length of subbasin slope (m), *slp* is the average slope of the subbasin (m/m), *n*_*ov*_ is the factor of Manning’s roughness in the subbasin, *v*_*c*_ is the medium channel speed (m/s), *area* is the surface of the subbasin (km^2^), *n*_*c*_ is the Manning factor for the canal and *slp*_*ch*_ is the canal slope (m/m). In the *Guadalest* basin, different *n*_*ov*_ coefficients were given to the land cover categories (Table 1) according to the Engman (1986) classes. Considering the experiences of Kehew et al. (2010) and Conesa et al. (2020), tested in similar Mediterranean semiarid catchments, main and tributary channels placed in subbasins with agricultural or natural land cover acquired a 0.065 value to *n*_*c*_ agreeing to the channel class of “Natural streams/Few trees, stones or brush” (Jin et al. 2000).

Evaporation losses from channels were obtained based on the following expression (Eq. (4)):

4 $${E}_{ch}={coef}_{ev} \bullet {E}_{o}\bullet {L}_{ch}\bullet W\bullet {fr}_{\Delta t}$$

where *E*_*ch*_ is the evaporation rate of the channel in a given day (m^3^ H_2_O), *coef*_*ev*_ is a channel evaporation adjustment factor, *E*_*o*_ is the potential evaporation (mm H_2_O), *L*_*ch*_ is the length of the channel (km), *W* is the canal width in view of water level (m) and *fr*_*∆t*_ is the time stage fraction with water flowing through the channel (Jin et al. 2000; Neitsch et al. 2011). According to Zhu et al. (2023) and Arnold et al. (2012), in ephemeral streams of the studied watershed, a 0.9 value was assigned to *coef*_*ev*_, since the numerical expression (Eq. (4)) uses to overestimate channel evaporation in semiarid and arid regions.

Transmission losses from the channels or streams to groundwater were estimated with Eq. (5):

5 $$tloss ={K}_{ch} \bullet TT\bullet {P}_{ch}\bullet {L}_{ch}$$

where *tloss* is the canal transmission loss (m^3^ H_2_O), *K*_*ch*_ is the effective hydraulic conductivity reached in the canal alluvium (mm/h), *TT* is the travel time of the flow (h), *P*_*ch*_ is the soaked perimeter (m) and *L*_*ch*_ is the canal distance measured in km (Samavati et al. 2023). Following our catchment knowledge, together with information from DPA-IGME (2009) and DGTP (2009), *K*_*ch*_ from main and tributary channels was fixed in the range of 6–25 mm/h corresponding to the materials characteristics of “Sand and gravel mixture with silt–clay content” (Lane 1983). The fraction of *tloss* that enters in the deep aquifer received a 0.2 value as stated by Arnold et al. (2012) in arid and semiarid watersheds.

#### Discharges from wastewater treatment plants

The *Guadalest* basin is close to the hydrologic natural regime (DPA-IGME 2015). The main anthropic alteration of its superficial regime corresponds to the *Confrides* WTP (Fig. 1), which pours its treated wastewater (108.3 m^3^/day) to the drainage network (CHJ 2023). In SWAT, this effluent was considered as “constant point source discharge” according to the Neitsch et al. (2011) nomenclature.

#### Setup of the SWAT model

After coupling previous data in SWAT, and solving the above equations for initial conditions, the model was run during the period with monthly stream gauge (1985–2015) to perform calibration and validation processes. Next, our calibrated SWAT code was executed with the following: (i) a daily scale to validate the achieved results with daily registers from aquifer springs throughout their available period (2007–2014) and (ii) a yearly scale to validate SWAT results with the SIMPA model (MIMAM 2000) during its annual modelled series (1980–2016). Also, due to the great meteorological variability of semiarid Mediterranean sites with high and concentrated rainfall episodes from convective systems (Jodar-Abellan et al. 2019b; Boix-Fayos et al. 2020), a warm up period (Winchell et al. 2013) of 5 years was fixed to all simulations.

### The SUFI-2 algorithm

Sensitivity analysis and later uncertainly test (validation and calibration) to right initial data of hydrological parameters, from SWAT, through stream gauge series (inverse modelling), were achieved with the *sequential uncertainty fitting version-2* (SUFI-2) available in SWAT-CUP (version 5.2.1.1: Abbaspour et al. 2015; Abbaspour 2019). This algorithm performs a stochastic calibration (parameter optimisation) where the initial parameter uncertainties, expressed as ranges or uniform distributions, are reduced through a cluster of iterations (usually 3 or 5 iterations with around 500 to 2000 simulations in each one). Goodness-of-fit assessment to observed data of stream gauges (flow discharges), sediments, nitrates, phosphates, etc. can be assessed based on results from the goal function and related statistics (Abbaspour et al. 1997). SUFI-2 has been effectively implemented in numerous cases globally (Gassman et al. 2014; Pandi et al. 2022; Aibaidula et al. 2023).

In this work, 17 hydrologic parameters, conditioned by the change methods of R-relative, V-replaces and A-absolute (Abbaspour 2019), were selected according to the following: (i) the watershed knowledge, based partially in previous studies (DGTP 2009; DPA-IGME 2015), (ii) results from the initial sensitive analysis (*P*-value $\le$ 0.05) and (iii) certain studies done in semiarid and arid karstic regions (Jodar-Abellan et al. 2018; Nguyen et al. 2020; Senent et al. 2020). Four iterations, of 2000 simulations each one, were run and tested through measured stream (monthly flow discharges) from the gauging station placed at the basin outlet (Fig. 1). This station belongs to the CEDEX (2023) network and depicts reliable streamflow series at monthly and yearly scale. Based on our objectives, validation was made with the 1985–1995 series and calibration with the 1996–2015 because this last period shows minimum data gaps. It should be noted that there are no other gauging stations from Spanish networks (ROEA 2023; SAIH 2023; SIA 2023; CHJ 2023; DPA-IGME 2015) in this watershed. The Nash–Sutcliffe efficiency, NSE, was selected as goal function since it is the main recommended statistic in long-term hydrologic studies, such as this study. NSE is one of the statistics that further improves the performance (adjustment) among simulations and observations (Nash and Sutcliffe 1970; Knoben et al. 2019; Jin et al. 2023). The other statistics chosen to test results from SUFI‐2 simulations were the *Kling-Gupta* efficiency, KGE, the percent of bias, PBIAS, the RMSE-observation standard deviation ratio, RSR and the *R*^2^ determination coefficient (Gupta et al. 2009; Krause et al. 2005; Moriasi et al. 2007). Moreover, a recently introduced statistical index, called Nash–Sutcliffe efficiency adjusted to arid regions, ANSE, was used to explore the SWAT aptitude to replicate observed stream data (Eq. (6)):

6 $$ANSE=1-\frac{{\sum }_{i=1}^{n} ({Y}_{i}^{obs}- {Y}_{i}^{sim}{)}^{4}}{{\sum }_{i=1}^{n} ({Y}_{i}^{obs}- {{\overline{Y} }_{i}^{obs})}^{4}}$$

where *Y*_*i*_^*obs*^ is the ith register of the assessed variable, *Y*_*i*_^*sim*^ is the ith simulation of this variable, ${\overline{Y} }_{i}^{obs}$ is the observation average and *n* is the number of total observations. ANSE depicts the same formulation of the classic NSE expression, except the exponent is increased from 2 to 4 to give more importance to extreme values. This change is important since in arid and semiarid regions, the standard NSE index usually overweights average values, and it cannot capture the hydrodynamic behaviour of almost zero values during long periods, jointly with eventual short and extreme values. Both statistics show the same ranges of variation (from 1 to − ∞), being 1 the best fitting. Detailed information about ANSE can be found in Valdes-Abellan et al. (2020) where it was effectively applied in Mediterranean semiarid regions.

### The SIMPA method

The integrated model to simulate precipitation-yield conversion (SIMPA) is a distributed rainfall-runoff method with spatial resolutions of 1 km × 1 km and monthly time steps (Estrela and Quintas 1996; MIMAM 2000; Ruiz 1998). This conceptual model solves water balance in each grid cell using a GIS database, built in GRASS (Álvarez et al. 2005), to calculate hydrological parameters from physical features of catchments (land cover, slopes, soil types, geology, etc.). In this method, the subsurface is separated into two parts: the soil moisture or superior non-saturated region and the inferior or aquifer area. This lower area is saturated and depicts groundwater reserves that might or might not be linked to the drainage system of the surface. Part of the precipitation input, with a monthly scale, remains into the soil humidity area, giving water to evapotranspiration also conditioned by temperature records from meteorological stations. The remaining rainfall may be stored as a surplus, flowing on the surface or recharging aquifers. Finally, surface runoff moves across the watershed in the current period, while infiltrated water is next discharged into the drainage system. SIMPA has been extensively applied to assess water reservoirs management and water availability from natural sources (Cabezas 2015; Gomariz and Alonso 2018; Pellicer and Martínez 2015), crop patterns and agricultural yields (Taguas et al. 2015), climate changes effects on water sources (Cakir et al. 2020; Estrela et al. 2012; UCLM 2005) and environment and water economy (Pedro-Monzonís et al. 2016; Vicente et al. 2016). Besides, recent developments of SIMPA are focused on flood events and quality assessment (SIMPA 2023). Currently, Spanish authorities (watershed districts among others) estimate national and regional water availability in natural regime (elements of the water balance as aquifer recharge, surface runoff, etc.) through this model (CEDEX 2023; CHJ 2023; SIMPA 2023).

In this study, results from SWAT (aquifer recharge, surface runoff and real evapotranspiration) were compared with the same variables from SIMPA to the period 1980–2016 at yearly scale. This spatial information was recovered from the most current SIMPA database of the Environment Spanish Ministry (SIMPA 2023). In this database, previous SIMPA spatial coverage has been improved: getting grids with 0.5 km × 0.5 km to the entire Spanish territory, considering more input data in SIMPA such as stations from the SIAR network, interpolating atmospheric variables, numerous improvements of the parameters used in the SIMPA calibration, etc. (SIMPA 2023). This spatial information on the Spanish territory was adapted to the boundary of the *Guadalest* basin through GIS-based tools.

### The recurrent neural networks (RNNs) algorithms

In the studied basin, from 2017 to 2050, groundwater recharge (GWR) series were built based on SWAT recharge results to the period 1980–2016. Recharge data from both periods were obtained with annual time steps and in each HRU. Among the current methods of deep learning, and especially considering the long-time series (sequential data) from this SWAT database, the recurrent neural networks (RNNs) algorithms are highly suitable to predict variables evolution (Li et al. 2023; Pandi et al. 2022), in this case annual groundwater recharge over 732 HRUs. Thus, this SWAT database was considered as input in three RNNs algorithms: the simple RNN, the gated recurrent unit neural network (GRU) and the long- and short-term memory neural network or LSTM (Alizadeh et al. 2022; Yang et al. 2023). In order to perform algorithms forecasting, first their simulation performance in real time was evaluated. Hence, for each HRU, GWR from 1980 to 1999 constructed a sequence of 20 inputs, and then GWR of 2000 was predicted. Then, the sequence of 20 inputs was moved 1 year forward (1981–2000), and the next coming year (2001) was predicted. This moving-forward procedure (Asadollah et al. 2023) continued until 2016 as the last target year under evaluation trial (using 1996 to 2015 as its input sequence). As in previous sections, NSE, KGE, PBIAS, RSR, *R*^2^ and ANSE were the statistics to test/validate recharge data obtained with the algorithms and the gained with SWAT from 2000 to 2016.

After algorithms validation, yearly GWR were forecasted over all 732 HRUs and to the period 2017–2050 (34 iterations) following a similar procedure to the predictive sequence on the real time. Uncertainty associated with this new period, without registers, was appraised through the widely established technique of 95 percent prediction uncertainty (95PPU) (Abbaspour 2019; Asadollah et al. 2024). This method depicts a specific range in which a forecasted value is highly expected to fall (Asadollah et al. 2021). To obtain this band, first, GWR across HRUs were averaged for each studied year between 2017 and 2050. Then, using the Monte-Carlo simulation method (Juncosa 1965), 1000 new GWR data was generated in each year and, afterward, sorted in ascending mode. Considering probabilities summation of all 1000 generated GWR data will equal to 1, and two samples with probabilities of 0.025 and 0.975 were extracted which respectively denotes the lower and upper uncertainty boundaries of the 95PPU (Yang et al. 2023; Asadollah et al. 2024).

Regarding the internal setting of the mentioned algorithms, in this work, these three methods were developed using the TensorFlow library of Python (Goh et al. 2023). Thus, each algorithm was applied using a semi-deep neural network (NN) with 3 hidden layers which shows internal nodes of 150, 100, and 50 units, respectively. These nodes were structured with the *tanh* layer as the activation function (Yang et al. 2023). In particular, the number of hidden layers and internal nodes were obtained using a trial-and-error procedure. For the training phase, 1000 epoch (with a corresponding batch size of 30) was found to be the good assumption in view of the number of inputs and the total sample points (Li et al. 2023; Yang et al. 2023). Also, tenfold cross-validation was used to evaluate the algorithms performance, meaning that in each iteration, 10% of data was kept out, while the models were put under training using the remaining 90%. Thus, the iteration process continued until all sample points were inputted in each algorithm as both training and testing samples (Goh et al. 2023). Detailed information of the workflow with the RNNs algorithms can be found as supplementary material of this manuscript.

### The exploitation index (EI) of groundwater

Aquifers’ state in the *Guadalest* watershed was assessed within the exploitation index (EI) during the period with registers (1980–2016) and throughout next decades (until 2050). Following the EI method, a groundwater formation (and/or a set of groundwater formations) suffers important pressures (overexploitation) if reaches EI greater than 0.8, beside an appreciable reduction of piezometric heights is recognised in a relevant portion of the groundwater formation (Vallejos et al. 2015; Derdour et al. 2023). In particular, this index encompasses groundwater extractions and the available resources stored in aquifers. More information about the EI, together with its main equation, can be found in CHJ (2015) and Jodar-Abellan et al. (2019a). In this basin, annual extractions to meet urban water demands were facilitated by local authorities during 1980–2016, while annual aquifers recharge estimated with SWAT and SIMPA for the same period was considered as the resource whose exploitation can be sustainable in line with Kalhor et al. (2019), Tohuami et al. (2013) and Cakir et al. (2020). Likewise, up to 2050, the EI was calculated with forthcoming groundwater recharge series, achieved with RNNs forecasting, and considering that aquifer extraction rates of the next years will continue similar to the current. This statement is justified as population inhabitants of the *Guadalest* catchment, supplied completely with these extractions, have scarcely increased during last decades (DGTP 2009; INE 2023).

## Results and discussion

### Sensitive and uncertainly test

The initial and global test of sensitivity, performed in our basin, was selected as sensitive parameters: GW_REVAP.gw, GWQMN.gw, GW_DELAY.gw and ALPHA_BF.gw from groundwater input files of SWAT (.gw), CN2.mgt from management (.mgt), r_SOL_AWC.sol and SOL_K.sol from soils (.sol), ESCO.bsn and FFCB.bsn from the basin inputs (.bsn), CANMX.hru and SLSOIL.hru from the hydrological response unit (.hru) and ALPHA_BNK.rte from the chief channel input (.rte). Complete information of these parameters can be found in Table 2 and in Arnold et al. (2012).

**Table 2:** Variation range of hydrological parameters in calibration: iterations 1 and 4 with 2000 simulations

Parameter	Description (unit)	Initial range used in calibration (iteration 1)	Fitted value (iteration 4)
r_CN2.mgt	Initial SCS (Soil Conservation Service) runoff curve number for moisture condition II (dimensionless)	− 0.1 to 0.1	0.09
v_CANMX.hru	Maximum canopy storage (mm H_2_O)	0 to 8	5.70
v_LAT_TIME.hru	Lateral flow travel time (days)	0 to 180	125.67
v_SLSOIL.hru	Slope length for lateral subsurface flow (m)	0 to 10	0.14
R_SOL_K.sol	Saturated hydraulic conductivity (mm/hr)	− 0.2 to 0.2	0.12
r_SOL_AWC.sol	Available water capacity of the soil layer (mm H_2_O/mm soil)	− 0.02 to 0.02	0.01
v_EPCO.bsn	Plant uptake compensation factor (dimensionless)	0.5 to 1	0.78
v_ESCO.bsn	Soil evaporation compensation factor (dimensionless)	0.2 to 0.9	0.74
v_FFCB.bsn	Initial soil water storage expressed as a fraction of field capacity water content (dimensionless)	0.0 to 1.0	0.85
a_CH_K1.sub	Effective hydraulic conductivity in tributary channel alluvium (mm/h)	− 0.1 to 0.1	− 0.07
a_CH_K2.rte	Effective hydraulic conductivity in main channel alluvium (mm/h)	− 0.1 to 0.1	− 0.07
v_ALPHA_BNK.rte	Baseflow alpha factor for bank storage (days)	0.0 to 1.0	0.32
v_ALPHA_BF.gw	Baseflow alpha factor (1/day)	0.0 to 1.0	0.43
a_GW_DELAY.gw	Groundwater delay time (days)	− 30 to 60	− 24.78
a_GWQMN.gw	Threshold depth of water in the shallow aquifer required for return flow to occur (mm H_2_O)	− 200 to 200	− 134.60
v_GW_REVAP.gw	Groundwater “revap” coefficient (dimensionless)	0.02 to 0.1	0.087
v_GW_SPYLD.gw	Specific yield of the shallow aquifer (m^3^/m^3^)	0.0 to 0.4	0.05

The presence of numerous groundwater parameters in the sensitive analysis depicted their great importance in the *Guadalest* watershed. Furthermore, in compliance with local experiences (DGTP 2009; DPA-IGME 2015) and international studies performed in arid and semiarid karstic areas (Nguyen et al. 2020; Al Khoury et al. 2023; Senent et al. 2020), parameters such as the shallow aquifer-specific yield (GW_SPYLD.gw) and lateral flow (LAT_TIME.hru) were considered in calibration (Table 2). Additionally, in basins, as in the study one, where evaporation and evapotranspiration processes are noticeably relevant (Gomariz et al. 2018), the plant uptake compensation factor (EPCO.bsn) must be encompassed in the calibration procedure. Particularly relevant was the ESCO parameter, selected in the sensitivity analysis, due to its important role in the evaporative demand abstraction of lower soil layers (Alcalá et al. 2018; Tan et al. 2020; Aloui et al. 2023). Finally, parameters related to the effective hydraulic conductivity of tributary and main channels (CH_K1.sub and CH_K2.rte) were added (Table 2) because of their relevance in this watershed (Palacios-Cabrera et al. 2022), the same as in numerous karstic basins around the world (Martínez and Conesa 2020; Nerantzaki et al. 2020; Senent et al. 2020; Nguyen et al. 2020).

Initial parameter ranges (iteration 1) and their last values afterward the calibration procedure (iteration 4, Table 2) were consistent with the pre-established domain of existence and in agreement with numerous works developed in semiarid and arid karstic catchments (Malagò et al. 2015, 2016; Mostofa-Amin et al. 2017). Final data for the chosen parameters were located approximately in the zone of higher probabilities, according to the pre-defined existence domain (Table 2). However, certain exceptions were identified. For instance, with the curve number for the humidity condition II (CN2 in Table 2), its final value is close to the higher range limit. From our knowledge, these results were coherent with the reality of the *Guadalest* watershed, which depicts steep slopes (around 30–40%) increasing the average curve number in the basin (Jodar-Abellan et al. 2019b). Similarly, the initial soil water storage (FFCB) with a value of 0.85 (Table 2) reflects the great capacity of the basin soils (Lithic Leptosols, Calcaric Cambisols, Regosols, etc.) to retain water (Ntona et al. 2023; Alcalá et al. 2018; Cakir et al. 2020). Furthermore, the parameter of groundwater “revap” (GW_REVAP) presents a high value (0.087) because of the great presence of forests in the watershed (see the “Studied watershed” section of the study). This encourages transmission of water to the root area and increases evapotranspiration rates (Molina et al. 2019). In contrast, final value of the groundwater delay time (− 24.8) is near the lower limit of its range (Table 2). Hence, in the SWAT model for this basin, values of groundwater delay among 5 and 6 days are identified, which are reasonable in a karst watershed of 62 km^2^ where water flows quickly, from the surface to the fractured underground through an interconnected network of conduits (Bailey et al. 2016; Mo et al. 2023).

**Fig. 3:**
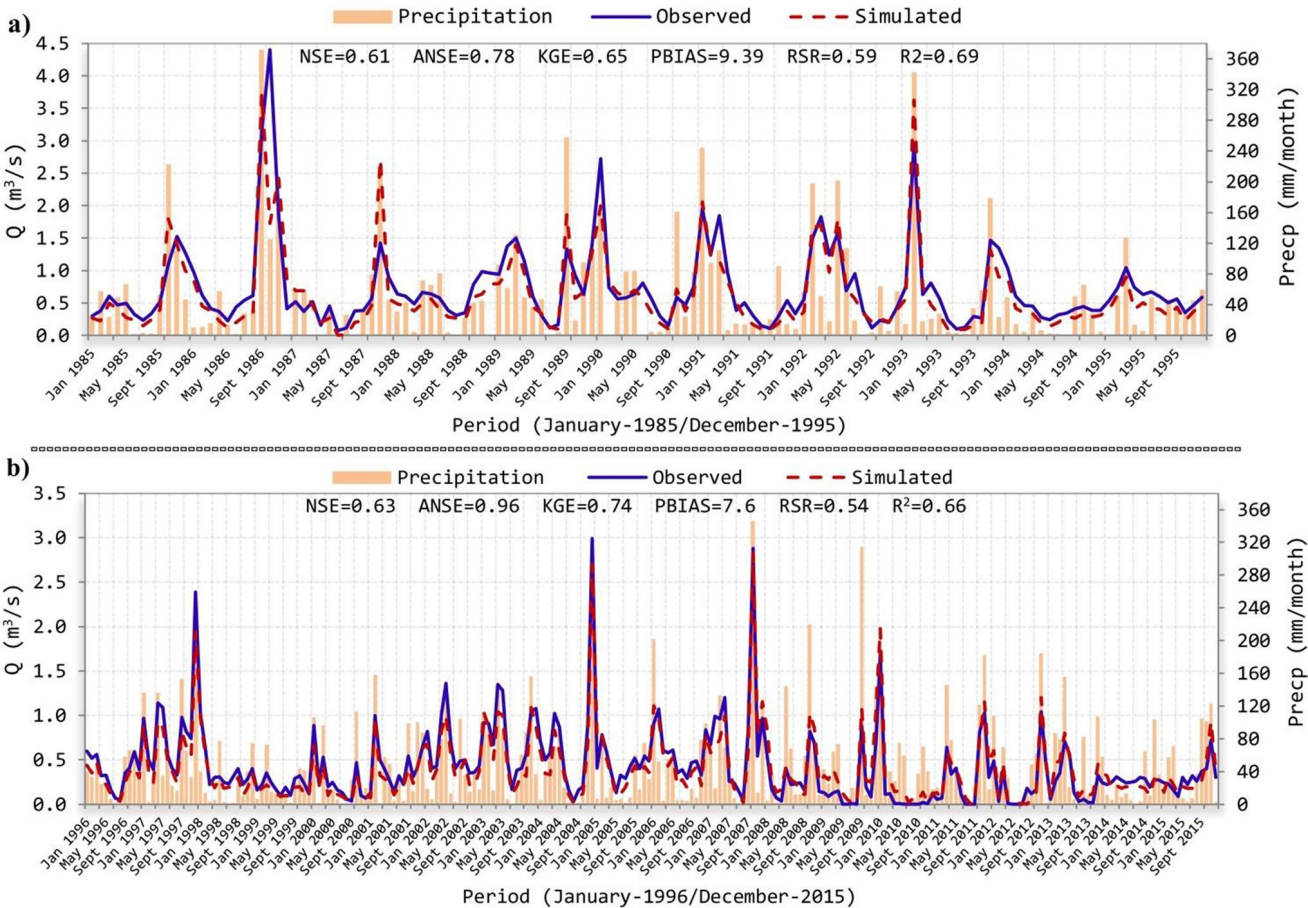
Validation (**a**) and calibration (**b**) fits with NSE as goal function

Final (fitted) values of hydrological parameters, achieved in the last iteration (Table 2), are contained in the best simulation of SUFI‐2, depicted in Fig. 3a to the validation stage (January 1985–December 1995) along with Fig. 3b for calibration (January 1996-December 2015). Both series show a suitable rainfall-runoff conversion considering the simulated streamflow regarding precipitation and streamflow measured records (Fig. 3). In addition, results from the considered statistical contrasts denote a correct fit taking into account the great streamflow variation proper of semiarid and arid Mediterranean zones (Fig. 3; Cabezas 2015; Vallejos et al. 2015). Thus, the NSE index acquires a value of 0.61 in validation and 0.63 in calibration, being both results classified as “satisfactory” agreeing with the statistic performance scores showed in Moriasi et al. (2007). The presented ANSE indicator improves the NSE results in validation (*ANSE* = 0.78) and in calibration (*ANSE* = 0.96) obtaining a consideration of “very good”. Essentially, this improvement is due to the greater consideration of extreme flow values by ANSE with respect to NSE, since the simulated and observed streamflow rates achieve a better convergence in these extreme values (Fig. 3). A “very good” result to the PBIAS index was identified in validation (*PBIAS* = 9.4%) but close to the limit (± 10%) stated in Moriasi et al. (2007), while a relevant enhancement was achieved in calibration (*PBIAS* = 7.6%). According to the PBIAS expression (Moriasi et al. 2007; Krause et al. 2005), both positives values of PBIAS represent a moderate underestimation of the observed flow during the study period.

Table 3 shows results of the considered statistical contrasts to the calibrated and uncalibrated SWAT model during validation and calibration. Generally, all statistics improve their behaviour in the calibrated model throughout both periods such as, for instance, the NSE and KGE indexes with values, respectively, of 0.61 and 0.65 during validation alongside 0.63 and 0.74 in calibration, close to their optimal range (Table 3). In particular, KGE the same as the ANSE index gives great importance to the extreme values of streamflow being, therefore, very relevant in sites with these features as semiarid karstic basins (Gupta et al. 2009; Abbaspour 2019). Continuing with the PBIAS analysis, this statistic suffers a distance from its optimal value (< ± 10%) in the uncalibrated model during validation (*PBIAS* = 24.8%, Table 3), although an optimal value was reached to this period in the calibrated SWAT (*PBIAS* = 9.4%), as well as during calibration (varying from − 5.5 to 7.6, Table 3). Despite most of these values are in the “optimal range” (PBIAS <  ± 10%), proposed by Moriasi et al. (2007), PBIAS behaviour informs that the medium estimated streamflow is moderately lower than the average observed streamflow in the calibrated model (Table 3). This is especially noted in the calibration period (January 1996-December 2015), where the simulated streamflow overestimates the observed measured record (*PBIAS* =  − 5.5) in the uncalibrated model, whereas in the calibrated SWAT a moderate underestimation is recognised (*PBIAS* = 7.6). Similar model performances comparing average values of simulated and observed flow were recognised in many arid and semiarid karstic basins due to great streamflow changes, conditioned in turn by the variable rainfall regime proper of these areas (Cabezas 2015; Malagò et al. 2016; Gomariz and Alonso 2018; Cakir et al. 2020).

**Table 3:** Statistical indexes achieved throughout monthly calibration and validation for streamflow

Statistical contrasts			Validation (Jan 1985-Dec 1995)		Calibration (Jan 1996-Dec 2015)	
Acron	Range	Opt. value	Uncalibrated	Calibrated	Uncalibrated	Calibrated
NSE	− ∞…1	1	0.08	0.61	0.24	0.63
ANSE	− ∞…1	1	0.61	0.78	0.75	0.96
KGE	− ∞…1	1	0.43	0.65	0.50	0.74
PBIAS	− 100%…100%	< ± 10	24.81	9.39	-5.48	7.6
RSR	0… + ∞	0	0.96	0.59	0.87	0.54
R^2^	0…1	1	0.61	0.69	0.60	0.66

**Table 4:** Average water balance variables (mm/year) in the *Guadalest* watershed during the validation and calibration periods

Variable (mm/year)	Validation (Jan 1985-Dec 1995)		Calibration (Jan 1996-Dec 2015)	
	Uncalibrated	Calibrated	Uncalibrated	Calibrated
Precipitation	619.8	619.8	566	566
Evaporation and transpiration	100.5	114.1	104.7	119
Surface runoff	149.6	107.7	109.1	76.8
Subsurface flow	72.1	103.3	68.9	96
Aquifer’s recharge	297.5	294.7	283.3	274.3

The above reduction of the simulated streamflow in the calibrated SWAT was a consequence of optimising certain parameters throughout SUFI-2 (Aibaidula et al. 2023; Jin et al. 2023). Thus, final values of parameters, such as max canopy content (CANMX), compensation coefficients of plant uptake (EPCO) and terrain evaporation (ESCO, Table 2), increased water retention from plant cover and soil layers, and thereafter, water was lost during evaporation and transpiration processes (Jimeno et al. 2022; Arnold et al. 2012). More differences among water balance variables, of the *Guadalest* catchment, during validation and calibration periods are shown in Table 4. In particular, a drier period was identified in the calibration set (January 1996-December 2015) with average rainfall amounts of 566 mm/year, while 620 mm/year were registered in validation (January 1985-December 1995). Similar precipitation trends were gathered in several semiarid and arid Mediterranean sites (Rouholahnejad et al. 2014; Taguas et al. 2015; UCLM 2005; Valdes-Abellan et al. 2017). As a result of the SUFI-2 optimisation (with CANMX, EPCO and ESCO among others), an increase of around 14 mm/year in evaporation and transpiration rates was reached along both study periods from the uncalibrated to the calibrated SWAT (Table 4). Hence, related elements of the water balance, as surface runoff, subsurface flow and groundwater recharge (Tan et al. 2020), receive less available water in the calibrated model: from 519.2 to 505.7 mm/year in the period January 1985-December 1995 and from 461.3 mm/year to 447.1 mm/year within January 1996-December 2015 (Table 4).

Fitted values for slope longitude of the lateral subsurface current (SLSOIL), and saturated hydraulic conductivity (SOL_K, Table 2), among others, caused a rise in the subsurface flow of around 30 mm/year in the calibrated model during both study periods. Therefore, decreases of surface runoff were estimated (Table 4). The obtained increase to the subsurface flow was coherent in a karst (fractured) area where surface runoff infiltrates across cavities of the terrain easily and, according to previous studies performed in the *Guadalest* watershed (DGTP 2009; DPA-IGME 2009), together with registered data of a developed vegetation cover in forest and agricultural land uses from the basin (see the “Studied watershed” section of the manuscript). Finally, aquifers recharge is approximately half (48%) of the total precipitation in the calibrated model (Table 4), which correctly represents a Mediterranean semiarid karst (fractured) watershed in line with similar studies (Tohuami et al. 2013; Vallejos et al. 2015; Ollivier et al. 2020).

### Assessing the water balance

#### Precipitation patterns

**Fig. 4:**
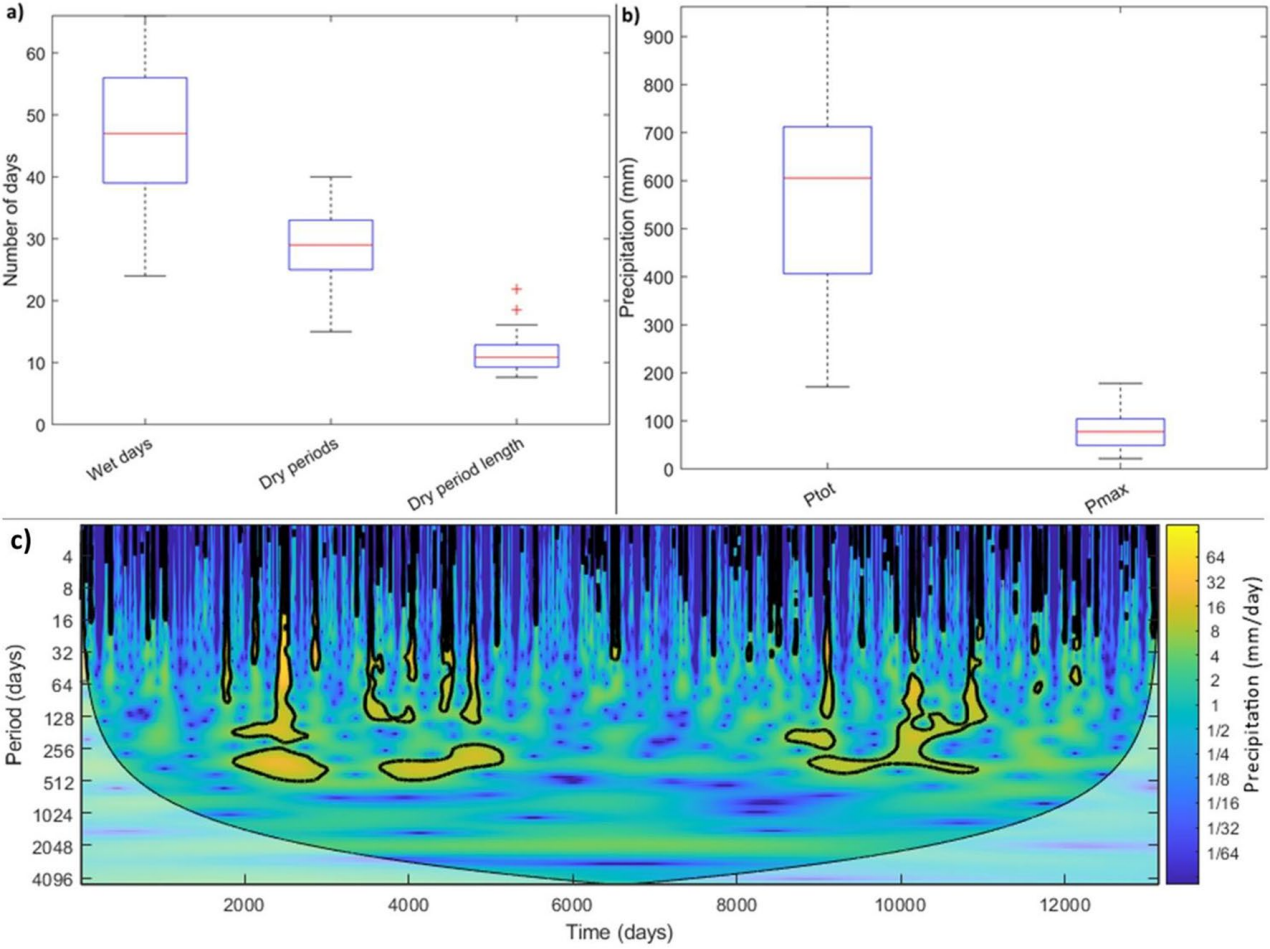
Box-whisker plots to the period 1980–2016 of **a** wet and dry periods, **b** maximum daily rainfall events (Pmax), and total annual rainfall (Ptot). The red segment, of the central part, shows the median; top box limits depict the 75th and 25th percentiles; the whiskers, encompassed until extreme points, do not reflect outliers, whereas outliers are drawn separately with a red “ + ” character. **c** Wavelet analysis of rainfall at daily scale during 1980–2016

Precipitation time series collected in the *Guadalest* watershed, during the period 1980–2016, depicted patterns characterised by long dry periods and short wet periods (Fig. 4a) typical of Mediterranean semiarid environments (Santos et al. 2017; Gil-Guirado et al. 2022). Above all, since median values of Fig. 4a, around 47 days per year were classified as wet days, while 28 dry periods/year were recognised, showing each dry period a length of 11.4 days (Fig. 4a). Therefore, 319 days/year were estimated as dry in the watershed. This substantial difference among the quantity of dry and wet days in our study area could increase during the next decades as stated in Valdes-Abellan et al. (2020), which identified that the climate of this region is changing to drier positions.

**Fig. 5:**
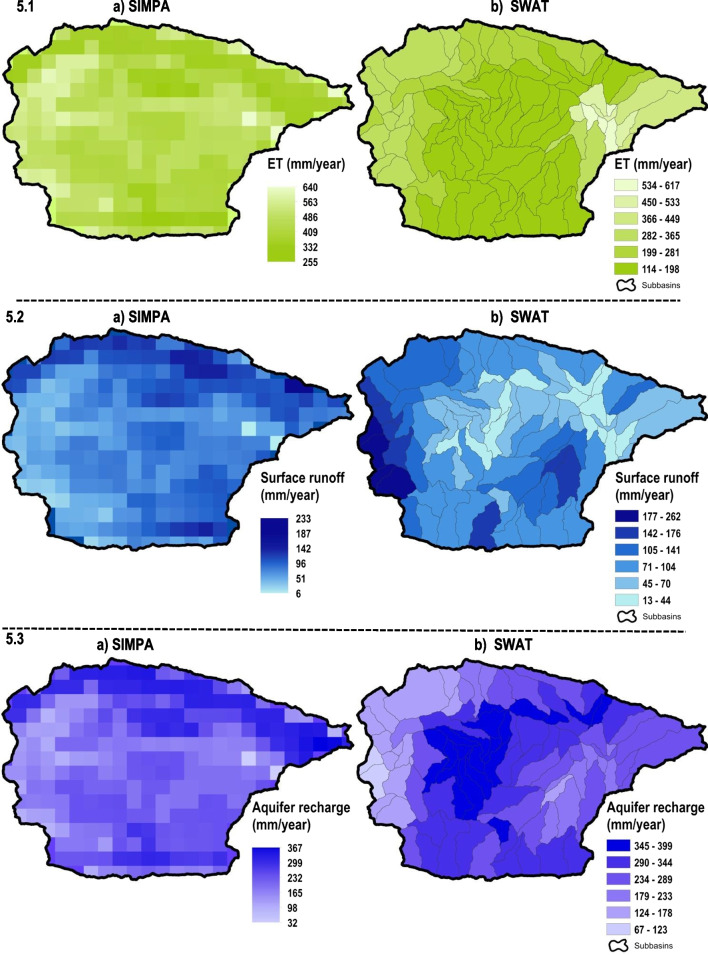
Annual rates of **5.1** real evapotranspiration, **5.2** surface runoff and **5.3** aquifers recharge in the *Guadalest* watershed through the a SIMPA and b SWAsT models during the period 1980–2016

It is worth to highlight that a reduced number of wet days per year encompass maximum daily precipitation events, which median value is close to 80 mm/day (Fig. 4b). These extreme events represent 14% of the total annual rainfall (above 600 mm/year) collected in the watershed (Fig. 4b). Thus, the performed wavelet analysis revealed that storm events are located mainly over September and October months, showing a remarkable frequency with significant cycles of 4 and 5 years (highlighted in Fig. 4c through precipitation “circles” of around 64 mm/day). This rainfall pattern is common in arid and semiarid Mediterranean watersheds which concentrate on high convective storms, especially during the conclusion of summer and throughout autumn (Sillero et al. 2021; Jodar-Abellan et al. 2019b; Gil-Guirado et al. 2022). These climate features depict relevant implications in the river and ravine regimes, which show long periods with very low or even absence of flow rates and a reduced number of periods when the almost completely annual flow volumes are concentrated (Palacios-Cabrera et al. 2022; Aloui et al. 2023).

#### Spatial distribution of real evapotranspiration, aquifer recharge and surface runoff

This section assesses average annual values of spatial distribution from surface runoff, real evapotranspiration and aquifer recharge. These processes, performed by the calibrated SWAT, were tested with the same results of the SIMPA method to the period 1980–2016.

Results of real evapotranspiration in the *Guadalest* basin are coherent between both considered models varying from 255 to 640 mm/year in case of SIMPA (Fig. 5.1a) and from 114 to 617 mm/year with SWAT (Fig. 5.1b). Therefore, a moderate underestimation of SWAT respect to SIMPA is identified regarding these extreme values. However, detail analysis denotes that, for example, just one grid cell of SIMPA presents the highest value (640 mm/year), which was located in the surface of the *Guadalest* reservoir (Fig. 5.1a and Fig. 1). Consequently, most of the grid cells in the watershed achieved similar values using SWAT or SIMPA.

Geographical analysis of real evapotranspiration revealed that the areas with higher altitude such as crests, crags and cliffs, obviously placed around the boundary of the catchment, depicted the lower values of real evapotranspiration (between 150 and 300 mm/year) in the two considered models (Fig. 5.1 a and b). This was because of both the great slopes (Fig. 1) and the sparsely vegetated areas (Fig. 2) identified in these mountain zones, which generate high surface runoff rates and difficult water retention from the scarce plant cover. Meanwhile, areas with less altitude and moderate slopes as in the valley bottom, thalwegs, riverbeds, etc., located in the central range of the *Guadalest* basin, showed great values of real evapotranspiration (among 500 and 640 mm/year) with SWAT and SIMPA. In both models, the highest rates (upper than 615 mm/year) were identified on the *Guadalest* reservoir corresponding to the estimated evaporation from this water surface (Fig. 5.1 a and b).

Similar evapotranspiration patterns were recognised in related studies realised in semiarid and arid Mediterranean sites. For instance, Valdes-Abellan et al. (2020), who studied climate change influences in the hydrological system of the *Mela* aquifer, obtained an average real evapotranspiration of 251 mm/year for their study period. These value results were coherent in line with the abovementioned range of 150 and 300 mm/year recognised in mountain zones of the *Guadalest* basin, where the *Mela* aquifer is located (Fig. 2). Hartmann et al. (2013) estimated real evapotranspiration values close to 200 mm/year in three major karst formations (southern Spain) by applying two karst hydrologic models (the reservoir model alongside the VarKarst tool). As well, in a karstic Mediterranean watershed of Crete (Greece), Nikolaidis et al. (2013) reached, with SWAT, an actual evapotranspiration of 455 mm/year.

In the *Guadalest* catchment, surface runoff varied from 6 to 233 mm/year and from 13 to 262 mm/year throughout the SIMPA and the SWAT models respectively (Fig. 5.2 a and b). Therefore, both models estimated similar surface runoff patterns in the watershed, although a moderate overestimation of SWAT regarding SIMPA is identified taking into account their lower and upper extreme values (Fig. 5.2 a and b). As stated previously in the real evapotranspiration analysis, minor differences among surface runoff extreme values are due to a reduced number of grill cells. Hence, the lowest value of surface runoff with the SIMPA model (6 mm/year) is identified in a cell placed on the *Guadalest* reservoir area, whereas the zone of this reservoir received surface runoff values among 13 and 15 mm/year with the SWAT model (Fig. 5.2 a and b). Besides, it is considered that the SWAT model estimated better upper values of surface runoff than SIMPA, because low surface runoff patterns were calculated in the southern and in the southwest portion of the basin by SIMPA (Fig. 5.2a). In particular, these areas with great steep slopes (Fig. 1) and permeability among low and very low (Fig. 2c) should depict high surface runoff values as the estimated by the SWAT model (Fig. 5.2b).

Numerous studies obtained comparable surface runoff results on Mediterranean semiarid and arid locations. For instance, Estrela et al. (2012), which employed the SIMPA code to appraise climate change effects on natural water resources from Spain, calculated mean actual runoff ranges sited between 26 and 200 mm per year in the northern part of the *Alicante* province, where the *Guadalest* basin is located, and among 0 and 100 mm/year to the rest of this province and close sites of the South of Spain. Jodar-Abellan et al. (2018), by using SWAT, estimated surface runoff rates of around 40 mm per year, during the period 2000–2010, in a Mediterranean basin placed in the south of the *Guadalest* watershed (in the SE Spain). In the European context, it should be mentioned in Nerantzaki et al. (2020) which simulated, through the SWAT code, an average surface flow of 262 mm/year during the period 1974–2018 in a Mediterranean karstic basin of Greece.

Aquifers recharge in the *Guadalest* watershed showed a range of 32 to 367 mm/year with the SIMPA model and from 67 to 399 mm/year using the SWAT model (Fig. 5.3 a and b). Therefore, values simulated by SWAT are 30–35 mm/year higher than values from SIMPA. However, as mentioned previously, differences in extreme values of the assessed water balance variables, usually, are due to a reduced cluster of grid cells.

Spatial distribution analysis of groundwater recharge with SIMPA denotes great recharge values in areas of the watershed with high altitude and great slopes, frequently placed around the basin boundary (Fig. 5.3a). However, SWAT calculated the highest groundwater recharge rates in the central portion of the *Guadalest* catchment where areas with less altitude and moderate slopes are identified (Fig. 5.3b). Thus, according to the field work performed in DPA-IGME (2009) and DGTP (2009) in the studied basin, and considering the achieved surface runoff patterns to this study area (Fig. 5.2 a and b), it is estimated that the SWAT model simulated aquifers recharge distribution more accurately than SIMPA in this watershed. In addition, similar trustworthy results of groundwater recharge by SWAT were obtained in Valdes-Abellan et al. (2020), where the KAGIS method was developed and applied in the *Mela* karstic aquifer (Fig. 2c).

### Assessing aquifer’s hydrological response

**Fig. 6:**
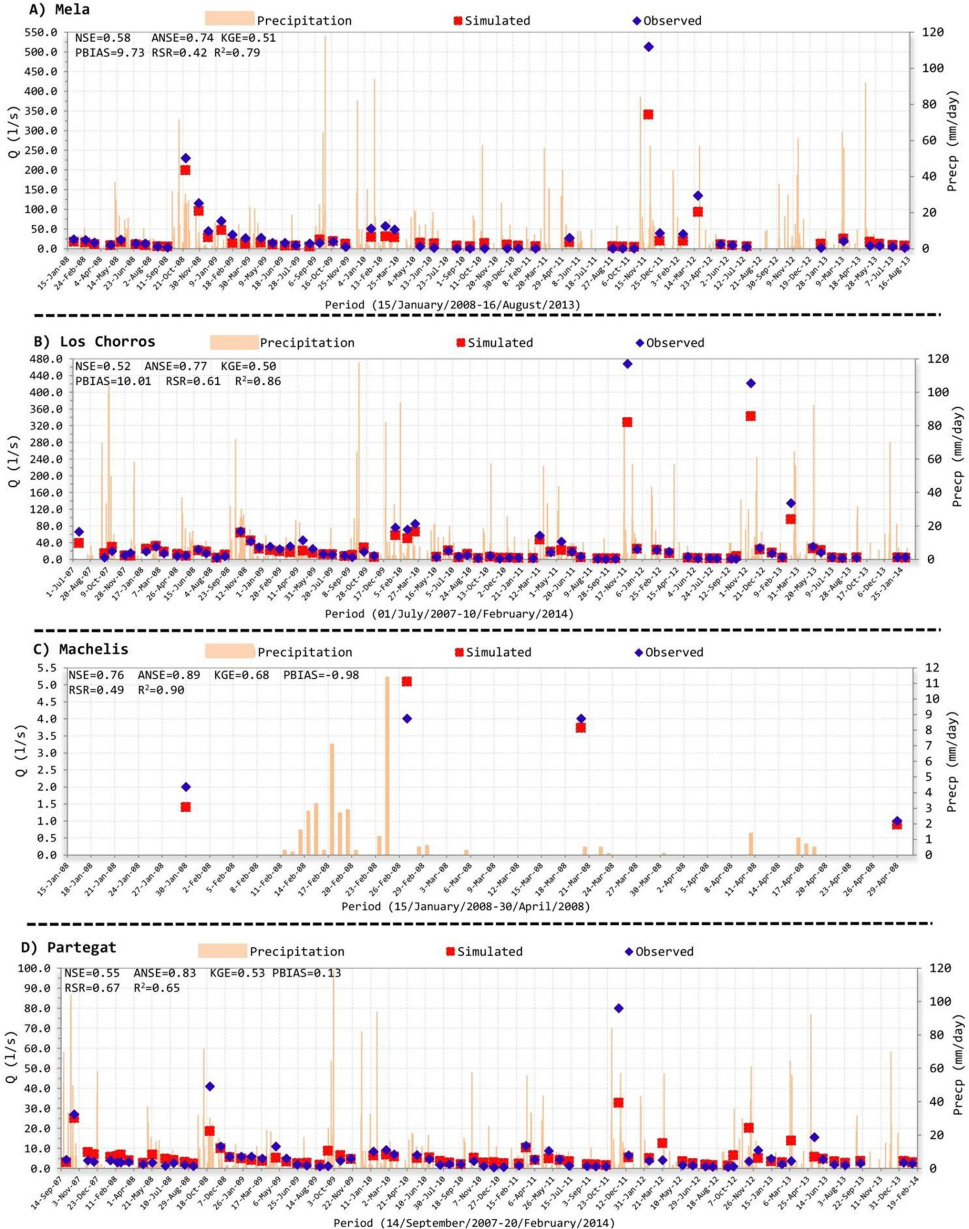
Observed and simulated groundwater flows in the *Mela* (**A**), *Los Chorros* (**B**), *Machelis* (**C**) and *Partegat* (**D**) aquifer springs

In this section, simulations of the groundwater contribution to the streamflow variable (Arnold et al. 2012), from the calibrated SWAT code, were tested against daily records of aquifer spring discharge facilitated by local authorities (CHJ 2023) for the period 2007–2014. This information belongs to the most important springs regarding the length of the natural groundwater discharges and the total volume of water, which are named as follows: *Mela*, *Los Chorros*, *Machelis* and *Partegat* (Fig. 1 and Fig. 6). Note that the model was not calibrated considering this hydrologic variable of aquifer discharges in springs, so the comparison of the simulated values with the real cases constitutes a strong test of the SWAT model.

The estimated aquifer discharge in springs depicts a rapid flow response to rainfalls collected in their close areas, as can be checked through simulated and observed flow data (Fig. 6). In simulations of the calibrated SWAT, this was possible because of fitted values of parameters such as GW_DELAY and GWQMN (Table 2). Both parameters control the response of groundwater discharge through the springs, and their values after the calibration process produced short groundwater delay and return flow processes respectively in the *Guadalest* basin, which results very reliable considering its well-developed karstic nature (DPA-IGME 2009; Hartmann et al. 2013; Tohuami et al. 2013; Vallejos et al. 2015).

*Mela* and *Los Chorros* groundwater springs showed “satisfactory performances” (Moriasi et al. 2007), among simulated and observed values, considering KGE and NSE statistics index (Fig. 6A and B). RSR obtained satisfactory fits in *Los Chorros* (*RSR* = 0.61) and very good results in *Mela* (*RSR* = 0.42) following Moriasi et al. (2007). Likewise, in line with Abbaspour et al. (2015), Krause et al. (2005) and Valdes-Abellan et al. (2020) statistics classification, ANSE, *R*^2^ and PBIAS depicted good and very good results for both aquifer springs (Fig. 6A and B). Goodness-of-fit measurements for *Machelis* and *Partegat* natural groundwater’s sources provided good results as the previous groundwater springs (Fig. 6C and D). In particular, in both cases, the entire considered statistics index received fits classified among “satisfactory”, “good” and “very good” (Moriasi et al. 2007; Gupta et al. 2009).

It is worthy to highlight that numerous studies accomplished in semiarid and arid Mediterranean zones with SWAT, and/or related methodologies, frequently do not present simulations tested (validated) with registers from aquifer springs due to their scarce and difficult accessibility (Jodar-Abellan et al. 2018; Mostofa-Amin et al. 2018; Senent et al. 2020), even more the use of discharge groundwater information for validation but not for the calibration process (which was developed using the flow rate in the watershed outlet). In the context of the karstic mountain area of the *Alicante* province, Valdes-Abellan et al. (2020) were the unique work whose outputs were calibrated and validated using groundwater discharge series. Results of that study reported an ANSE of 0.89 alongside a normalised root-mean-square error, NRMSE, equals to 0.06 for the 10-year-long calibration period. Both results are coherent with those obtained in this manuscript for the *Mela* aquifer using a longer time series in the present work (Fig. 6A).

### Assessing current and expected groundwater availability to supply urban water demands

#### At catchment scale

SWAT series of groundwater recharge (GWR) to the period 1980–2016, at yearly scale and over the 732 HRUs achieved in the *Guadalest* basin, were coupled with three RNN algorithms (the simple RNN, the gated recurrent unit neural network or GRU and the long- and short-term memory neural network or LSTM) to estimate recharge evolution from 2017 to 2050.

Table 5 depicts algorithms validation between its predicted recharge data and the obtained with SWAT from 2000 to 2016. Attending to performance ratings from Moriasi et al. (2007), the three algorithms reached accuracies considered as “good” and “very good”. Thus, the LSTM reported best accuracy in the six statistical contrasts evaluated (Table 5) showing some indexes results close to their optimal value (*R*^2^ = 0.99, *NSE* = 0.94, *PBIAS* = 0.5 among others) and, therefore, “very good” classifications in the six cases. As expected, the GRU was the second algorithm with better results (*R*^2^ = 0.86, *NSE* = 0.81, *PBIAS* = 0.9) and the simple RNN the worst (*R*^2^ = 0.79, *NSE* = 0.76, *PBIAS* = 1.4; Table 5). In line with Li et al. (2023), although the three considered algorithms encompass self-looping mechanisms which allow the participation of preceding steps in the foraging of forthcoming steps, LSTM is the unique which shows the addition of memory cells as well as input, forgot and output gates (Asadollah et al. 2021). This fact along with its structure, composed by four special layers which simultaneously authorise the exclusion of useless information and understand correlations and trend between data sequences (Li et al. 2023), explains the great performance rate reached with the LSTM algorithm during validation (2000–2016). Likewise, despite this advance type of neural network is extremely popular in issues regarding the gradient explosion and disappearance that usually present samples with long sequences (Goh et al. 2023), it has been scarcely coupled and tested with well-established hydrological models, the same as the simple RNN algorithm and the GRU (Alizadeh et al. 2022; Pandi et al. 2022). From the author’s knowledge, just Yang et al. (2023) implemented these types of algorithms and verified them with hydrological tools. In that study, placed in the *Dagu* watershed (China), predicted runoff by algorithms was compared with SWAT results, reaching suitable performance ratings among both methods (*RMSE* = 16.5, *MAE* = 8.3, *R*^2^ = 0.68).

**Table 5:** Statistical indexes obtained during algorithms validation at yearly scale and over 732 HRUs of SWAT

Statistical contrasts			Validation (2000**–**2016)		
Acron	Range	Opt. value	Simple RNN	GRU	LSTM
NSE	− ∞…1	1	0.76	0.81	0.94
ANSE	− ∞…1	1	0.86	0.82	0.95
KGE	− ∞…1	1	0.68	0.77	0.96
PBIAS	− 100%…100%	< ± 10	1.4	0.9	0.5
RSR	0… + ∞	0	0.93	0.74	0.11
R^2^	0…1	1	0.79	0.86	0.99

**Fig. 7:**
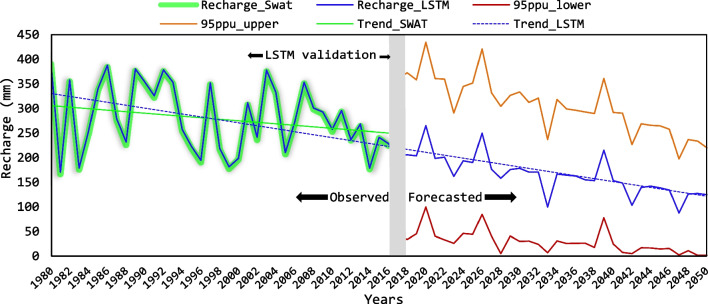
Annual groundwater recharge rates from 1980 to 2050 in the *Guadalest* watershed

Figure 7 shows yearly groundwater recharge as an average of all HRU of the watershed and throughout the period 1980–2050. Along with SWAT, LSTM was selected to represent GWR evolution as it offered best results in algorithms validation (Table 5). It should be highlighted the decreasing recharge trend reached with both methods from 1980 to 2016 (Fig. 7). This issue can be confirmed with recharge rates obtained with SWAT from 1985 to 1995 (295 mm/year) and the gained by the model from 1996 to 2015 (274 mm/year; Table 4). During the LSTM forecasted period (2017–2050), this trend continued decreasing (Fig. 7). In 2050, for instance, an average recharge of 125 mm/year was predicted which surged to 220 mm/year in view of the upper 95PPU boundary and decreased to 2 mm/year within the lower 95PPU. Therefore, each of these three scenarios depicted lower recharge rates than the average gained between 1980 and 2016 (278 mm/year with SWAT and 280 mm/year with LSTM; Fig. 7). Recharge reduction rates during the coming decades, together with great decreases of several hydrological variables, have been widely predicted in many scientific studies globally (Estrela et al. 2012; Pandi et al. 2022; Yang et al. 2023). In the southeast of Spain, Jodar-Abellan et al. (2018) estimated an average recharge reduction of 16% during the period 2021–2050 in the *Taibilla* karst watershed. Similarly, in the *Mula* and *Algeciras* basins, Martínez et al. (2021) predicted available water resources decreases among 52% and 56% over the next decades. Therefore, these forecasts are close than the obtained in the current study with predicted recharge decays of around 50% in 2050 (Fig. 7).

#### At aquifer scale

The six towns located in the *Guadalest* basin are supplied mainly with water resources from the *Mela*, *Beniardá-Polop*, *Benimantell* and *Serralla-Aixorta* aquifers (DPA-IGME 2015). Table 6 summarises annual extractions achieved by these small localities for the period 1980–2016. Based on aquifer recharge results, estimated as the average of SWAT and SIMPA (Fig. 5.3 and Table 6) on a yearly scale for the same period, water inflows are higher than extractions, and therefore, urban water demands depict a good guarantee of groundwater supply except in the *Beniardá-Polop* aquifer where extractions exceed groundwater recharge (7.1 hm^3^/year and 4.96 hm^3^/year, respectively). Thus, this overexploited aquifer receives an exploitation index (EI) of 1.43, being the limit among the good and bad groundwater states placed at 0.8 (CHJ 2015; Derdour et al. 2023).

**Table 6:** Extractions, groundwater recharge and their relation within the exploitation index (from 1980 to 2050)

Aquifer	Extraction (hm^3^/year) (1980–2016)	Recharge (hm^3^/year)			Exploitation index		
		DPA-IGME (2015)(1980–2012)	SWAT & SIMPA (1980–2016)	LSTM (2017–2050)	DPA-IGME (2015)(1980–2012)	SWAT & SIMPA (1980–2016)	LSTM (2017–2050)
Mela	0.04	0.3	0.21	0.14	0.13	0.19	0.29
Beniardá-Polop	7.1	5.65	4.96	4.22	1.26	1.43	1.68
Benimantell	0.09	0.41	0.36	0.19	0.22	0.25	0.47
Serralla-Aixorta	0.08	-	0.42	0.31	-	0.19	0.26

Likewise, the aquifers recharge results published by DPA-IGME (2015) are close to the estimated in this work by the average of SWAT and SIMPA. However, a moderate underestimation of both models is recognised (Table 6). To the best of our knowledge, this underestimation can be attributed to the length of the study periods due to DPA-IGME (2015) collected data up to 2012, while the present work involves data until 2016 (Table 6). In the *Ventós-Castellar* karstic aquifer, placed near the *Guadalest* basin towards the south, similar recharge amounts were obtained by Tohuami et al. (2013) which collected data until 2008. Therefore, because in the south-eastern Spain recent years are shifting to drier positions (Palacios-Cabrera et al. 2022), available water to recharge aquifers is moderately lower in the present study than in DPA-IGME (2015) or in Tohuami et al. (2013). Consequently, between 1980 and 2016, the EI calculated with aquifers recharge from SWAT and SIMPA were higher in the four cases than those achieved in DPA-IGME (2015) until 2012 (Table 6). As well, these results are in agreement with the EI calculated by Jodar-Abellan et al. (2019a) throughout 12 groundwater bodies placed near the *Guadalest* watershed and towards the south. In that study, the most exploited groundwater formations were *Sierrra de Argallet* with an EI of 1.63 and *Sierra de Crevillente* with an EI of 3.38, while lower exploitation indexes were found in *Sierra Aitana* (*EI* = 0.18) and in *Agost-Monnegre* (*EI* = 0.13).

According to the algorithm with best statistical accuracy, the LSTM (Table 5), forecasted recharge until 2050, will reduce in the four aquifers (Table 6). Thus, the *Mela* aquifer will suffer an average recharge reduction of 33.3% (from 0.21 hm^3^/year during the period 1980–2016 until 0.14 hm^3^/year over 2017–2050), whereas decreases of 14.9%, 47.2% and 26.2% were predicted respectively in the *Beniardá-Polop*, *Benimantell* and *Serralla-Aixorta* formations (Table 6). Regarding the *Mela* aquifer, equivalent recharge rates were projected by Valdes-Abellan et al. (2020) under current climate conditions (0.26 hm^3^/year) and within global change scenarios (− 14% in RCP2.6 and − 22% in RCP8.5).

In line with previous results, over the next decades, exploitation indexes will surge in the four aquifers (Table 6). Hence, an increase of 52.6% was expected in the *Mela* aquifer (from an EI of 0.19 over the period 1980–2016 until an EI of 0.29 throughout 2017–2050). As well, the *Beniardá-Polop*, *Benimantell* and *Serralla-Aixorta* formations achieved, respectively, an EI of 1.68 (+ 17.5%), 0.47 (+ 88%) and 0.26 (+ 36.8%; Table 6). During the period 2041–2070, similar trustworthy findings were acquired by Estrela et al. (2012) in the *Júcar* river basin district, which involves the *Guadalest* watershed, with an expected increase of the water exploitation index quantified in 87.8%.

It is noted that the previous exploitation indexes, up to the period 2017–2050, were reached assuming that current aquifer extractions (Table 6) will continue at similar rates in the coming decades. This can be assumed since the number of inhabitants served by the studied aquifers has remained stable in recent years (INE 2023) and considering that urban water supply is the unique anthropic usage of these formations (DPA-IGME 2015). However, in case of aquifer extractions rise and available water to recharge aquifers continues to decrease over the coming decades, as stated in these areas by Valdes-Abellan et al. (2017) and Jodar-Abellan et al. (2018) due to a predicted reduction of annual precipitations along with several increases of evapotranspiration rates; the above-mentioned aquifers will reach an overexploitation context (*EI* > 0.8). Hence, new sources of water resources are needed to guarantee the supply of urban water demands. In addition, DPA-IGME (2015) found that most of water, which recharges these karstic aquifers, is drained in the following days through natural springs, fissures, large pipes and conduits from the subsoil to the terrain surface. A large percentage of this water volume is then removed due to processes such as evaporation, retention by the soil profile and vegetation cover (CHJ 2015; Vallejos et al. 2015; Cakir et al. 2020). Thus, in the assessed basin, it should be highlighted that a moderate percentage of the volume recharging the aquifers is available to supply nearby municipalities.

### Limitations of the study

The present manuscript depicts groundwater forecasting especially exhaustive through the four aquifers with registers (*Mela*, *Beniardá-Polop*, *Benimantell* and *Serralla-Aixorta*). However, this basin is composed by other groundwater formations currently without reliable time-series data to be considered as inputs in hydrological models. This fact, alongside the absence of a complete Spanish cartography of aquifers (a common situation in numerous countries), provides a relevant limitation to this scientific study. Following Xue et al. (2024), in future works, remote sensing sources will be assessed in order to compensate the lack of groundwater information in this and in related watersheds of the Spanish Mediterranean arc.

## Conclusions

Principal findings achieved in this work are presented below:

(i) Implemented high-resolution SWAT method, along with GIS-based tools, has demonstrated to be correct modelling a semiarid karstic Mediterranean catchment with partial lack of groundwater information. In this context, and within the SWAT code, the curve number method to the humidity condition II besides the Muskingum model was appropriate to simulate precipitation-runoff transformation and flood hydrodynamics respectively. Hence, overland and channel Manning expressions, regarding flow speed, alongside the Manning roughness factors selected, were relevant to convert the real land cover classes of the assessed watershed into SWAT and flow velocity in both the main channels and the overland flow.
(ii) Through the accomplished sensitivity and uncertainly analysis with monthly streamflow (validation and calibration processes), the selected hydrologic parameters along with their final values improved the SWAT performance. Thus, values of the selected statistics measuring the goodness of fit (NSE, KGE, PBIAS, RSR, etc.) were categorised as good, very good and satisfactory. Even better results were achieved for the new proposed statistic index (ANSE) which reported a final result close to its optimal limit (1) within the calibration procedure. Goodness-of-fit measurements for validation become not as good as the obtained in calibration but in satisfactory ranges on a general trend.
(iii) An extra validation for the calibrated SWAT model was tested with the following: (i) aquifers spring discharges in the *Guadalest* watershed, getting trustworthy results in the considered statistical contrasts, especially within the ANSE index and (ii) simulations from the Spanish national hydrologic modelling performed in this country with the distributed SIMPA model. Both models, SWAT and SIMPA, depicted coherent results in the real evapotranspiration patterns, although SWAT estimated more properly than SIMPA variables such as surface runoff and aquifers recharge.
(iv) Obtained satisfactory results within the open-source SWAT code are useful, especially in a difficult basin to be modelled due to its geological features (karstic domain) along with its climate conditions, characterised by long dry periods and short wet periods. In addition, extreme rainfall events are located in a reduced number of these wet days per year.
(v) Among the considered recurrent neural networks used to forecast groundwater recharge in the basin until 2050, the LSTM reported best-performance results during algorithms validation. Principally, this was due to its great ability to deal with sequence-based datasets. According to LSTM predictions, groundwater recharge will continue decreasing in the coming decades and, after 2040, even with reductions greater than 50% compared to current recharge levels. As a consequence, the exploitation indexes of the four aquifers with registers will increase considerably.
(vi) Local authorities, responsible for water management, could consider current results from SWAT and SIMPA together with algorithms forecasting in the assessed and in related karstic Mediterranean catchments. In particular, both models result allow solving several issues identified in the evaluated basin such as water resources available to supply nearby populations, showing an aquifer problems of exploitation with extractions that surpass water recharge in the current period. Based on our analysis, this context of groundwater exploitation will extend in the watershed during next decades. Moreover, the SWAT tool will permit performing of further research related to water quality problems and agricultural land use abandonment.

### Supplementary Information

Below is the link to the electronic supplementary material.

Supplementary file1 (DOCX 26 KB)

Supplementary file1 (DOCX 15.0 KB)

## Data Availability

The data will be provided upon a reasonable request to the corresponding author.
