# Supplementary material for: Assessing current and future available resources to supply urban water demands using a high-resolution SWAT model coupled with recurrent neural networks and validated through the SIMPA model in karstic Mediterranean environments
Source: Environ Sci Pollut Res Int. 2024 Jul 24;31(36):49116–40. doi: 10.1007/s11356-024-34404-5 (PMC11310254; doi:10.1007/s11356-024-34404-5)
Supplement: Supplementary file 1 — Supplementary file1 (DOCX 26 KB) [file 11356_2024_34404_MOESM1_ESM.docx]

**Supplementary material: detail setting of the Recurrent Neural Network (RNN) algorithms**

The workflow followed in the present work, with the RNN algorithms, is described below:

1. The forgot gate, which usually uses sigmoid activation functions, evaluated the historical data (yearly aquifers recharge from 1980 to 2016 over 732 HRUs) and decided which sample need to be excluded from the previous cell called $C_{t-1}$ (Li et al., 2023; Yang et al., 2023). This also involves that the $f_{t}$ expression (Eq.1) equals to either of 1 or 0 as the result of the preceding output $(h_{t-1})$ and the current input $(x_{t})$. In particular, be equal to 0 or 1 denotes the matter of dealing with previous information as the former deal with retraining while the latter indicates the forgetting (Asadollah et al., 2021).

|  | $f_{t}=\sigma(W_{f}\left[ x_{t},h_{t-1} \right]+b_{f})$ | (1) |
| --- | --- | --- |

1. The information passed to a memory cell was performed in two stages. According to Alizadeh et al. (2022), initially the input gate decides which $f_{t}$’s from equation (1) will be used for updating. This new nominee vector, called as $i_{t}$ (Eq.2), created the subsequent information, represented by $\tilde{C_{t}}$, by applying a layer with the $tanh$ activation function (Eq.3).

|  | $i_{t}=\sigma(W_{i}\left[ x_{t},h_{t-1} \right]+b_{i})$ | (2) |
| --- | --- | --- |
|  | $\tilde{C_{t}}=tanh(W_{c}\left[ x_{t},h_{t-1} \right]+b_{c})$ | (3) |

1. Based on the produced information in the above stages, the *f_t_*, the $C_{t-1}$, the *i_t_* and the $\tilde{C_{t}}$ elements were involved in a new variable (*C_t_*) as shown in the following expression (Eq.4).

|  | $C_{t}=f_{t}\times C_{t-1}+i_{t}\times\tilde{C_{t}}$ | (4) |
| --- | --- | --- |

1. In order to decide which of this new obtained information would pass as the output, two major steps were also required (Asadollah et al., 2021). Like in previous stages, a $o_{t}$ was created in similar fashion using a sigmoid layer (Eq.5). Then, the newly generated $o_{t}$ was multiplied with information obtained from passing the $C_{t}$ through a $tanh$ layer. This product will form the final output (*h_t_*) of the current cell (Eq.6).

|  | $o_{t}=\sigma(W_{o}\left[ x_{t},h_{t-1} \right]+b_{o})$ | (5) |
| --- | --- | --- |
|  | $h_{t}=o_{t}\times tanh(C_{t})$ | (6) |
