# Supplementary material for: Assessing current and future available resources to supply urban water demands using a high-resolution SWAT model coupled with recurrent neural networks and validated through the SIMPA model in karstic Mediterranean environments
Source: Environ Sci Pollut Res Int. 2024 Jul 24;31(36):49116–40. doi: 10.1007/s11356-024-34404-5 (PMC11310254; doi:10.1007/s11356-024-34404-5)
Supplement: Supplementary file 2 — Supplementary file1 (DOCX 15.0 KB) [file 11356_2024_34404_MOESM2_ESM.docx]

**Author’s information**:

1.-Dr.Antonio Jodar-Abellan, PostDoc researcher. [*ajodar@cebas.csic.es*](mailto:ajodar@cebas.csic.es) Links:

<https://www.researchgate.net/profile/Antonio-Jodar-Abellan>

<https://publons.com/researcher/5003735/antonio-jodar-abellan/>

<https://www.scopus.com/authid/detail.uri?authorId=57203890014>

<https://orcid.org/0000-0003-3373-8952?lang=es>

2.-Dr.Miguel Ángel Pardo. Full professor. [mpardo@ua.es](mailto:mpardo@ua.es). Links:

<https://orcid.org/0000-0001-8779-3258>

<https://publons.com/wos-op/researcher/1301546/miguel-angel-pardo-picazo/>

<https://www.researchgate.net/profile/Miguel-Pardo>

<https://www.scopus.com/authid/detail.uri?authorId=57193165508>

<https://cvnet.cpd.ua.es/curriculum-breve/es/pardo-picazo-miguel-angel/45185>

3.-Seyed Babak Haji Seyed Asadollah. PhD researcher. shajiseyedasadollah@esf.edu. Links:

<https://scholar.google.com/citations?user=qmBKSu0AAAAJ&hl=en>

<https://www.researchgate.net/profile/Babak-Hs-Asadollah>

4.-Dr.Ryan T. Bailey. Full professor. Email: [RyanT.Bailey@colostate.edu](mailto:RyanT.Bailey@colostate.edu). Links:

<https://orcid.org/0000-0002-6539-1474>

<https://jtcox94.wixsite.com/rtbailey>

<https://www.scopus.com/authid/detail.uri?authorId=26032500800>

<https://www.researchgate.net/profile/Ryan-Bailey-7>
